# Proteome analysis develops novel plasma proteins classifier in predicting the mortality of COVID‐19

**DOI:** 10.1111/cpr.13617

**Published:** 2024-02-25

**Authors:** Yifei Zeng, Yufan Li, Wanying Zhang, Huidan Lu, Siyi Lin, Wenting Zhang, Lexin Xia, Huiqun Hu, Yuanlin Song, Feng Xu

**Affiliations:** ^1^ Department of Infectious Diseases Second Affiliated Hospital of Zhejiang University School of Medicine Hangzhou China; ^2^ Shanghai Key Laboratory of Lung Inflammation and Injury, Department of Pulmonary Medicine, Zhongshan Hospital Fudan University Shanghai China; ^3^ Key Laboratory of Multiple Organ Failure (Zhejiang University) Ministry of Education Hangzhou China; ^4^ Research Center for Life Science and Human Health Binjiang Institute of Zhejiang University Hangzhou China

## Abstract

COVID‐19 has been a global concern for 3 years, however, consecutive plasma protein changes in the disease course are currently unclear. Setting the mortality within 28 days of admission as the main clinical outcome, plasma samples were collected from patients in discovery and independent validation groups at different time points during the disease course. The whole patients were divided into death and survival groups according to their clinical outcomes. Proteomics and pathway/network analyses were used to find the differentially expressed proteins and pathways. Then, we used machine learning to develop a protein classifier which can predict the clinical outcomes of the patients with COVID‐19 and help identify the high‐risk patients. Finally, a classifier including C‐reactive protein, extracellular matrix protein 1, insulin‐like growth factor‐binding protein complex acid labile subunit, E3 ubiquitin‐protein ligase HECW1 and phosphatidylcholine‐sterol acyltransferase was determined. The prediction value of the model was verified with an independent patient cohort. This novel model can realize early prediction of 28‐day mortality of patients with COVID‐19, with the area under curve 0.88 in discovery group and 0.80 in validation group, superior to 4C mortality and E‐CURB65 scores. In total, this work revealed a potential protein classifier which can assist in predicting the outcomes of COVID‐19 patients and providing new diagnostic directions.

## Graphical abstract

The study collected the plasma from COVID‐19 patients to find differentially expressed proteins between the death and survival groups and constructed a five‐protein classifier in relation with the early prediction of the mortality within 28 days of COVID‐19 patients' admission.

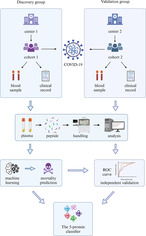

## 1 INTRODUCTION

Corona Virus Disease 2019 (COVID‐19) has been global public health issues in past 3 years, bringing a great burden to the world medical system.^1^ According to numerous epidemiological investigations,^2^ most of patients with COVID‐19 show mild symptoms and good prognosis and recover health without therapies or with minor treatments, therefore, they are classified as mild and moderate types.^3^ However, some patients have acute respiratory distress syndromes and need instant therapies, including oxygen treatment, non‐invasive ventilation and emergency endotracheal intubation,^4^ so they are classified as severe and critical types. Chinese guidelines of diagnosis and treatment of the novel coronavirus infection (the 10th version) point out the old with serious comorbidities will be easier to develop into severe/critical and showed higher mortality. The previous studies showed, from 1st September to 26th December 2022, Chinese cursory aggregate severe/critical case rate was 0.035%. In Shanghai Public Health Center, 0.35% patients without comorbidities and 2.68% patients with comorbidities developed into severe/critical.^5^ Up to 1st February 2023, the Chinese Center for Disease Control and Prevention reported cumulative 81,560 deaths, most of whom were the old with serious comorbidities, suggesting that COVID‐19 is particularly harmful to the elderly population.

Recently, XBB has become the main virus strain in Chinese mainland, taking up a proportion of 92.4% from 22nd to 28th May 2023. It is estimated 2777 new cases and 164 new deaths were reported in May, with an average age of 79.3 years old. Due to the high severe/critical case rate and mortality among the elderly population, it was necessary to construct a plasma‐protein classifier which can assist in diagnosis and early prediction of the patients' prognosis.

Proteomics mainly explores the consists and changes of different proteins in cells, tissues and bodies. In recent years, proteomics has been applied in various aspects of clinical medicine and also contributes much in virological researches. For example, previous researches revealed novel Zika virus host factors,^6^ mechanisms of dengue and Zika virus pathogenesis,^7^ antiviral factors and viral evasion mechanisms,^8^ metabolic strategy for herpesvirus replication^9^ and independent risk factor of the prognosis of sepsis^10^ by utilizing proteomics.

The precise identifications of proteins and polypeptides make it unique advantages in COVID‐19 studies, which can overcome the problems brought by the degradation of proteins and improve the accuracy of researches. For instance, West Lake University disclosed the differential expressions of sera proteins of COVID‐19 patients and discovered proteins involved in platelet degranulation were significantly downregulated in severe patients.^11^ Besides, the research from London University found plasma proteomic features could reflect the discrepancies among the inflammation reactions related to severity and duration of the symptoms.^12^ Jinyintan Hospital also identified several biomarkers which could distinguish the prognosis of COVID‐19.^13^ In addition, a joint research also developed a machine learning model of sera proteins based on the pre‐vaccination condition to predict the production and maintenance of antibodies after vaccination.^14^

In this study, we collected plasma from 277 COVID‐19 patients in the discovery group at different time points after their admissions and 97 COVID‐19 patients in the validation group within 24 h of their admissions and set the mortality within 28 days of patients' admissions as the clinical outcome. We divided the whole patients into death and survival groups and explored the changes of plasma proteins during the course of the disease between the two groups in discovery group. Finally, we found a group of plasma proteins which differentially expressed between survival and death groups during the course of disease and developed a plasma‐protein classifier with five differentially expressed proteins (DEPs). The protein classifier can predict the clinical outcome of the patients using trace plasma collected within 24 h of patients' admissions, identifying potentially high‐risk patients among them and instructing further managements. Besides, we also compared the five‐protein classifier with other conventional biomarkers and efficient prediction scores, further demonstrating its superior practicability in clinical practice.

## 2 METHODS

### 2.1 Participants and clinical data collection

The research was approved by the hospital ethics committee. We collected plasma samples from 277 patients and 97 patients with COVID‐19 (positive for nucleic acid detection test or antigen test) in the Second Affiliated Hospital, Zhejiang University School of Medicine and Zhongshan Hospital, Fudan University respectively, from December, 2022 to January, 2023. Then, we stored them at −80°C freezer. For discovery group from the Second Affiliated Hospital, we collected plasma samples from different time points (the 1st day, the 3rd day, the 7th day, 14th day and the 21st day) in the course of the disease, up to the 21st day after the admission. For validation group from Fudan Hospital, we collected the plasma on their first day of admission (within 24 h). The whole patients in the discovery group and validation group were further divided into death and survival groups which were used to carry out the subsequent analysis. The severity of patients was identified by Chinese guidelines of diagnosis and treatment of novel coronavirus infection (the 10th version). This study excluded patients with autoimmune diseases, long‐term chemotherapy or hormone therapy, bone marrow or peripheral blood stem cell transplantation and human immunodeficiency virus infection.

The clinical data were from electrical medical records, including gender, age, comorbidities, C‐reactive protein (CRP), white blood cells (WBC) count, neutrophil‐lymphocyte ratio (NLR), neutrophil and lymphocyte counts, the length of stay (LOS) and 28‐day mortality. Besides, we also collected the 4C Mortality score which is created by International Severe Acute Respiratory and emerging Infections Consortium World Health Organization, using sign measurements [Respiratory rate (RR) ≥20/min and Glasgow coma scale score <15], laboratory findings (peripheral oxygen saturation on room air <92%, urea >7 mmol/L and C‐reactive protein ≥50 mg/L), gender, comorbidities and age to predict mortality,^15^ and Expanded‐CURB65 (E‐CURB65) score system which includes eight indicators: age ≥65 years, Lactic Dehydrogenase (LDH) >230 U/L, Albumin <3.5 g/dL, Platelet count <100 × 109/L, Confusion, Urea >7 mmol/L, RR ≥30/min, low systolic (<90 mmHg) or diastolic (≤60 mmHg) blood pressure.^16^

### 2.2 Preparation of peptides

We extracted peptides from the plasma samples with some modifications.^11^, ^17^ Briefly, 1 μL of plasma from each specimen was lysed using 30 μL lysis buffer (8 M urea in 100 mM ammonium bicarbonate, ABB) at 32°C for 30 min. The plasma proteins were then reduced with 10 mM tris (2‐carboxyethyl) phosphine (TCEP) and alkylated using 40 mM iodoacetamide (IAA). We added 110 μL of 100 mM ABB in the samples to dilute the urea before the enzymatic digestion. Then, the protein extracts were digested at 32°C with a two‐step tryptic digestion, an enzyme‐to‐substrate ratio of 1:60 (final ratio 1:30), for 4 and 12 h, respectively. We adjusted the pH to 2–3 using 1% trifluoroacetic acid (TFA) to stop the digestion. Next, peptides were cleaned with SOLAu columns (Thermo Fisher Scientific, San Jose, USA) to prepare for MS analysis.

### 2.3 SWATH‐MS analysis

We injected peptides of the samples to an Eksigent NanoLC 400 System (Eksigent, Dublin, CA, USA), companied with a TripleTOF 5600 system (SCIEX, CA, USA) for the SWATH‐MS analysis as previously described.^17^ Five hundred nano gram of peptides for each sample was separated along an analytical column (3 μm, ChromXP C18CL, 120 Å, 150 × 0.3 mm) with a 20 min LC gradient of 5%–30% buffer B, at a flow rate of 5 μL/min. For the SWATH method used in this study, we set a 55 variable Q1 isolation window scheme. The accumulation time was set to 100 and 31 ms for the MS1 scan and the MS/MS scan, respectively. The duty time per cycle was 1.8 s.

### 2.4 Proteome data analysis

For proteomic data analysis, we first generated an experiment‐specific subset library (subLib) using priori analyses of the SWATH data^17^, ^18^ containing 8431 precursors and 1224 protein groups from the Swiss‐Prot database of Homo sapiens. In the DIA‐NN settings,^19^ the quantification strategy was set as robust LC (high accuracy) and other parameters were all default. Protein and peptide false discovery rates were set not to exceed 1%.

### 2.5 Quality control

The quality of proteomic data was ensured at multiple levels. First, plasma samples of discovery and validation cohorts were randomly distributed in 26 and 4 different batches, respectively. A pooled plasma sample combined from all samples of discovery cohort was prepared for each batch, and 45 pairs of biological replicates from the same plasma sample were designed to control the quality during protein digestion. After digestion, a pooled peptide sample was prepared and run per batch to control the quality during MS acquisition. Fifty‐two samples were injected twice as technical replicates.

Pearson correlation coefficients among pooled samples, and those between each pair of biological replicates and technical replicates were calculated using protein intensity, respectively (Figure S1). All of them were above 0.8, but one pair of biological replicates (Cr3A12). Thus, we deleted the one with the lower numbers of quantified proteins between biological replicates of Cr3A12. Then, we performed the log2 transformation of protein intensity, and removed batch effects by batch and MS machine using Limma R package (Ver. 3.44.3). Batch effects were evaluated using Principal Component Analysis.

### 2.6 Pathway/network analysis

Student's *t* test was performed for 28‐day survival and death groups by week 1, 2, 3, respectively, using the discovery dataset of 622 samples collected from day 2 of admission and thereafter. Adjusted *p* values were calculated using B‐H correction. Fold change (FC) was calculated by the mean of proteins intensity between these two groups. The criteria for significantly DEPs were that the adjusted *p* value should be less than 0.05 and FC should be larger than 1.5.

The pathway enrichment was analysed by Metascape web‐based platform^20^ (with *p* value <0.05), or string web‐based platform^21^ for total DEPs.

### 2.7 Machine learning

XGBoost (Ver. 1.7.4) was used in Python (Ver. 3.9) to generate a machine learning model using the discovery dataset of 88 samples collected at the admission time (t0, namely the first 24 h within patients' admission). First, we randomly divided the dataset into a training dataset (*n* = 58) and an internal test dataset (*n* = 30) for five‐fold cross‐validation. Fifty‐six DEPs were filtered aforementioned and six immunoglobins among them were excluded for feature selection. Two parameters, namely learning rate (from 0.01, 0.05, 0.1 to 0.3) and max_depth (from 1 to 8 with step at 1), were optimized using left 50 DEPs. Those with the maximum values of the mean area under the curves (AUCs) in internal test dataset among five‐fold cross‐validation were selected for the following importance rank. Next, the top five protein features by importance were chosen to make up the final classifier. The two foregoing parameters were optimized again using these five features. The final model was generated with learning rate of 0.1 and max_depth of 1 using the discovery dataset (including the training dataset and the internal test dataset). The threshold for predictive score was set as 0.2 according to the proportion of samples between two groups. The performance of the final machine learning model was evaluated in an independent validation cohort from the other centre.

### 2.8 Statistical analysis

Receiver operating characteristic (ROC) curve was used to evaluate the AUCs of the existing biomarkers (age, CRP, lymphocyte, neutrophil and NLR) and score systems (the 4C mortality and E‐CURB65 scores) for predicting the expected outcome. The value at the maximum Youden index of each biomarker was seemed as the cut‐off value. GraphPad Prism 9.5.0 (GraphPad software) was used to analyse the data. All the tests were two tailed and the results were regarded as statistically significant when *p* value <0.05.

## 3 RESULTS

### 3.1 Clinical characteristics of the patients

**FIGURE 1:**
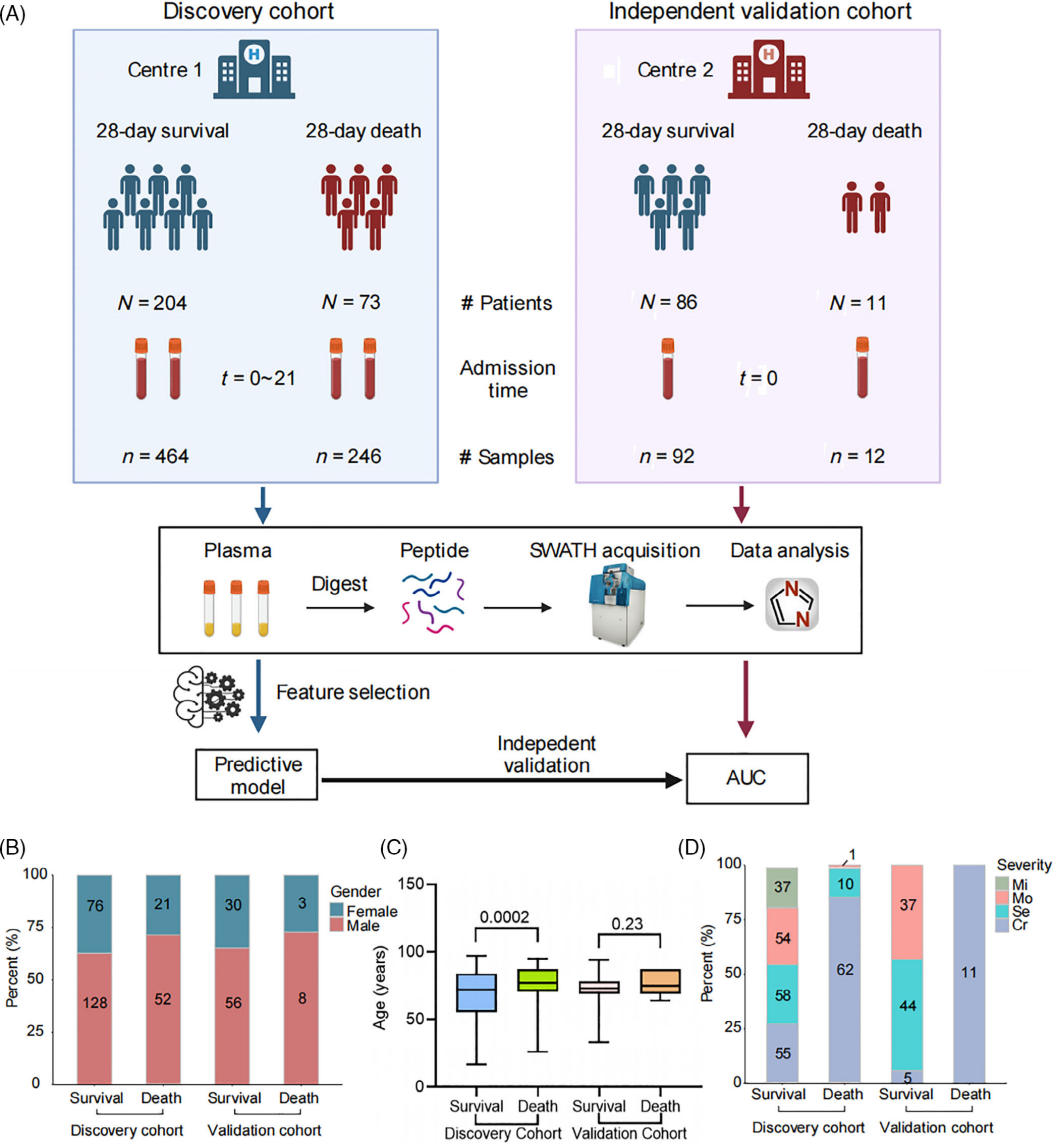
Study design and patients. (A) Overview of blood samples collection from COVID‐19 patients, including the discovery cohort from centre 1 and the validation cohort from the other centre. The workflow for processing the proteomic data was shown, including the plasma separation, protein digestion, LC–MS/MS analysis, database search and further computational analyses. (B) The gender distribution between 28‐day survival and 28‐day death groups of the discovery cohort and independent validation cohort. (C) The age distribution. Data points indicate the data of single patient at each time point and are presented as median with interquartile range. The centre line within each box shows the median, and the top and bottom of each box represent the 75th and 25th percentile values, respectively. (D) The severity distribution.

For discovery cohort, after 28‐day follow‐up, 204 patients were determined to be survivors (including patients still in hospital and discharged with better conditions) and 73 patients were confirmed as dead. The plasma samples were calculated as 464 survivals and 246 deaths (including biological replicates). There were 76 females (37.3%) and 128 males (62.7%) of survivors, including 37 mild (18.1%), 54 moderate (26.5%), 58 severe (28.4%) and 55 critical (27%) cases. The 52 males (71.2%) and 21 females (28.8%) death patients covered 1 moderate (1.4%), 10 severe (13.7%) and 62 critical (84.9%) cases. Besides, the median age of death patients was significantly greater than that of survival patients (*p* < 0.001). For validation group, there were 86 survival and 11 death patients from Zhongshan Hospital. Thirty female (34.9%) and 56 male patients (65.1%) were involved in the survival group, covering 37 moderate (43%), 44 severe (51.2%) and 5 critical (5.8%) cases. Three dead female (27.3%) and 8 dead male patients (72.7%) were all critical patients (Figure 1). The complete features of the two cohorts are shown in Table 1.

**TABLE 1:** The characteristics and outcomes of patients enrolled in discovery and validation groups.

Characteristics	Discovery group (*n* = 277)	Validation group (*n* = 97)
Demographic characteristics		
Age (years)	74 (17–97)	73 (33–94)
males	180 (64.98)	64 (65.98)
Comorbidities		
Hypertensive heart disease	136 (49.10)	60 (61.86)
Diabetes	66 (23.83)	40 (41.24)
Chronic obstructive pulmonary disease	26 (9.39)	10 (10.31)
Liver disease	8 (2.89)	9 (9.28)
Coronary artery disease	44 (15.88)	18 (18.56)
Renal dysfunction	17 (6.14)	7 (7.22)
Congestive heart failure	31 (11.19)	18 (18.56)
Cerebrovascular disease	34 (12.27)	23 (23.71)
Neoplastic disease	33 (11.91)	14 (14.43)
Prehospital treatment	193 (69.68)	92 (94.85)
Laboratory findings		
CRP (mg/L)	59.3 (17.45–115)	44.1 (8.7–69.95)
WBC (10^9^/L)	6.9 (4.65–9.75)	7.23 (5.03–9.64)
Neutrophils (10^9^/L)	5.48 (3.53–8.69)	5.1 (3.4–7.95)
Lymphocytes (10^9^/L)	0.66 (0.43–1.00)	0.9 (0.5–1.3)
NLR	8.19 (4.14–16.98)	5.71 (3–12.9)
4C mortality score class		
0–3	44 (15.88)	3 (3.09)
4–8	40 (14.45)	19 (19.59)
9–14	151 (54.51)	59 (60.82)
≥15	42 (15.16)	16 (16.50)
E‐CURB65 score class		
0–2	94 (33.94)	38 (39.18)
3–4	120 (43.32)	47 (48.45)
5–8	63 (22.74)	12 (12.37)
Clinical outcomes		
28‐day mortality	73 (26.35)	11 (11.34)

*Note*: Data are presented as median (interquartile range) or *n* (%).

### 3.2 Plasma proteomics analysis of patients with COVID‐19

**FIGURE 2:**
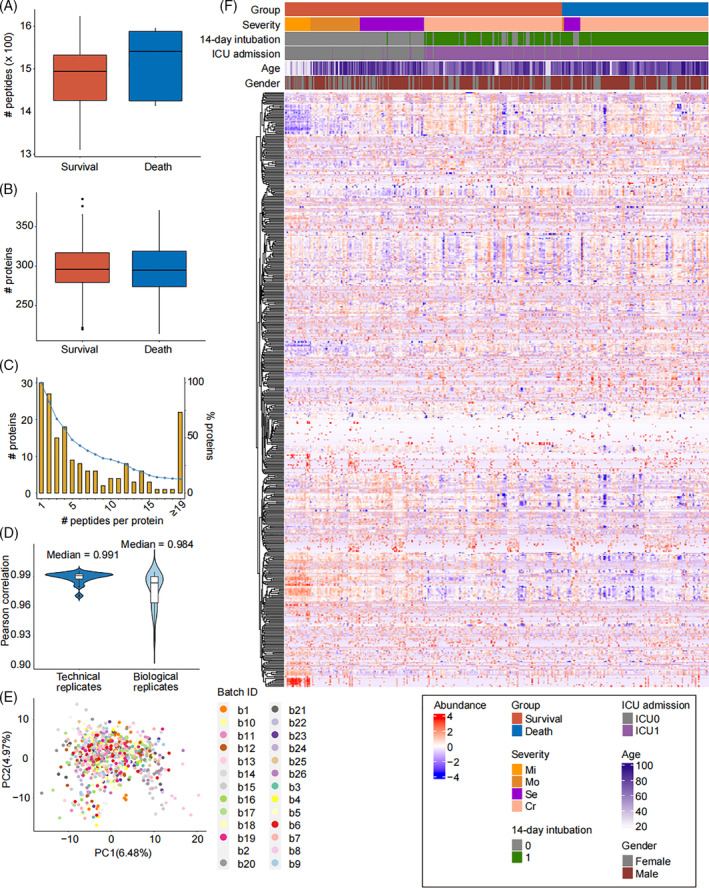
Proteome profiling of plasma samples from COVID‐19 patients of discovery cohort. (A,B) The distribution of numbers of quantified peptides and proteins between 28‐day survival and 28‐day death groups of the discovery cohort. (C) The distribution of peptide numbers of quantified proteins. The orange bar represents protein count with relevant number of peptides. The blue curve represents accumulative proportion of proteins from those with more than 19 peptides per protein to one peptide. (D) Pearson correlation coefficients between each pair of technical replicates and biological replicates from discovery cohort. (E) Unsupervised clustering of whole proteomic data among different batches of discovery cohort. (F) The heatmap of proteomic data of the discovery cohort.

We extracted peptides from the plasma samples in discovery group and filtered qualified peptides for the next analyses. These peptides were then mapped to corresponding protein sequences to quantify proteins. The distributions of the quantified peptides and proteins between survival and death groups from discovery group have been presented in Figure 2A,B, showing the number of proteins was equal between the two groups. Then, the distribution of peptide numbers of quantified proteins was explored (Figure 2C). To control the quality of the proteomic data, we used protein intensity to calculate Pearson correlation coefficients between each pair of biological replicates and technical replicates and found the median coefficients were respectively 0.984 and 0.991 (Figure 2D), indicating the reliability of the results. In total, 530 peptides and proteins were obtained for following researches. Moreover, principal‐component analysis (PCA) was performed to classify 530 proteins and found they were mainly divided into 26 clusters (Figure 2E). Finally, 530 reserved proteins were analysed utilizing the hierarchical clustering method to visualize the results in a heatmap (Figure 2F). The results showed numerous proteins were differentially expressed in different groups of plasma samples (survival and death, male and female, mild, moderate, severe and critical and ages), implying possible prediction proteins can be distinguished from the proteomic data.

### 3.3 The changes of plasma proteins between survival and death groups

**FIGURE 3:**
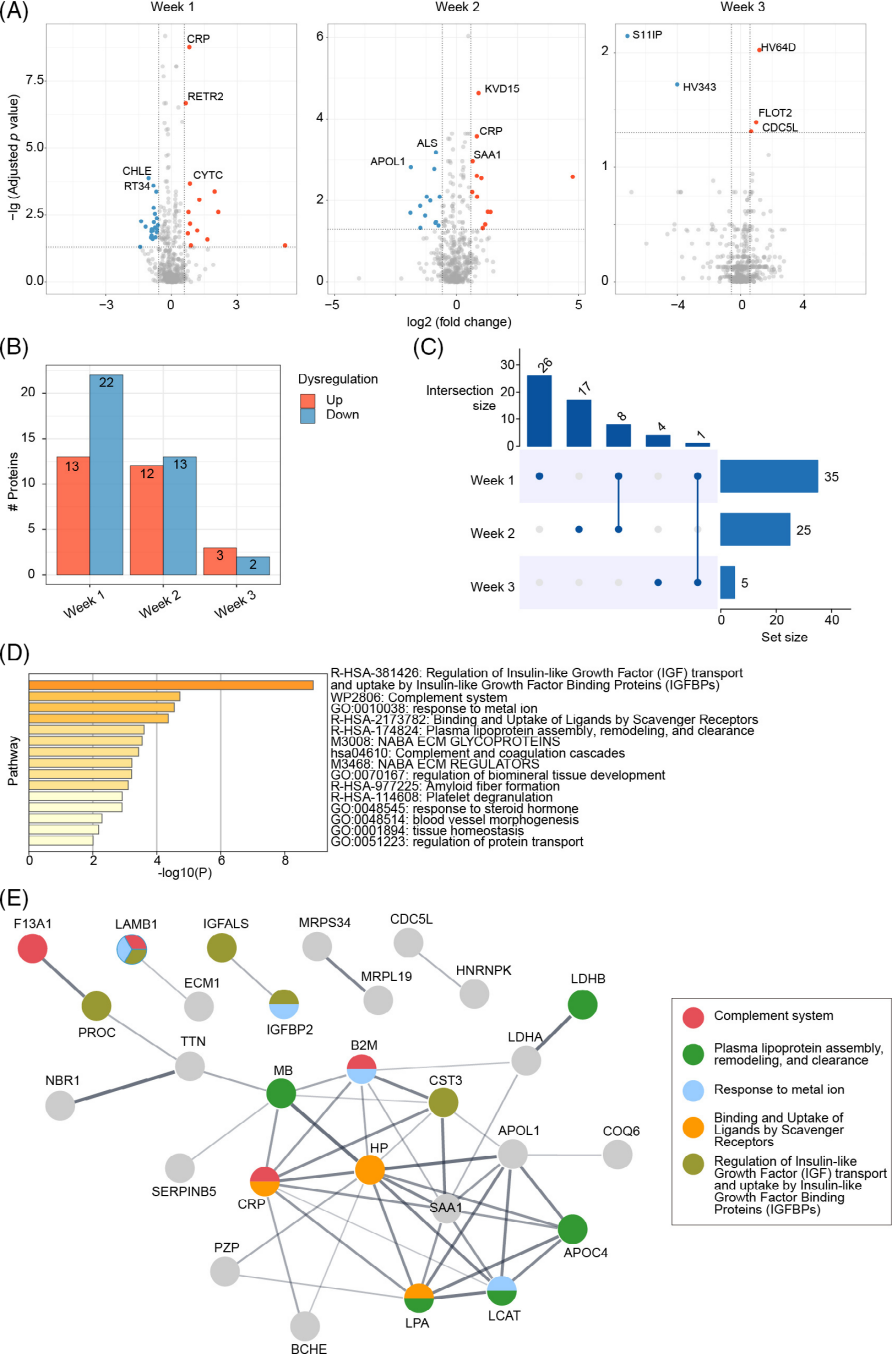
Proteomic alternations between 28‐day survival and 28‐day death groups of the discovery cohort. (A) Dysregulated proteins of plasma collected at week 1, 2 and 3 between two groups. The top five proteins with most significant B‐H adjusted *p* value were labelled with protein names. Fold change represents protein intensity of 28‐day death versus 28‐day survival. (B) Summary of numbers of dysregulated proteins. (C) The overlapping of dysregulated proteins among three times frames. (D) Enriched pathways for overall dysregulated proteins using Metascape. (E) The network of dysregulated proteins by String and their involved functions.

Then, we explored the DEPs from week 1, 2 and 3 between survival and death groups and screened top 5 DEPs with the most significant B‐H adjusted *p* value. The results showed for the first week, CRP, reticulophagy regulator 2 and Cystatin‐C were the most up‐regulated proteins while cholinesterase and small ribosomal subunit protein mS34 were the most down‐regulated proteins in the death group. In the second week, immunoglobulin kappa variable 3D‐15, CRP and serum amyloid A‐1 protein were the most up‐regulated when insulin‐like growth factor (IGF)‐binding protein complex acid labile subunit and apolipoprotein L1 were the most down‐regulated in the death group. For the third week, immunoglobulin heavy variable 3‐64D, flotillin‐2 and cell division cycle 5‐like protein were the most up‐regulated as serine/threonine‐protein kinase 11‐interacting protein and immunoglobulin heavy variable 3–43 were the most down‐regulated in the death group (Figure 3A). According to the analysis, the differences between the two groups were the most significant in the first week, displaying 13 up‐regulated and 22 down‐regulated proteins. There were 12 up‐regulated and 13 down‐regulated proteins in the second week and 3 up‐regulated and 2 down‐regulated proteins in the third week (Figure 3B). We also explored the overlaps among the DEPs in the 3 weeks and found eight DEPs were the same in the first and second weeks. However, there was only one protein shared between the first and third week as no protein was shared between the second and third weeks (Figure 3C). Therefore, 56 DEPs in total were determined using the plasma samples collected among the 3 weeks.

Afterwards, pathway enrichment analysis was carried out on the DEPs by Metascape web‐based platform (Figure 3D). The result showed regulation of IGF transport and uptake by IGF binding proteins (IGFBPs) obtained the highest enrichment ratio scores. Besides, complement system, assembly, remodelling and clearance of plasma lipoprotein and binding and uptake of ligands by scavenger receptors were also highly enriched, indicating the severity of COVID‐19 was connected to acute inflammations and dysregulations of macrophages and lipid metabolism, which is in accordance with previous studies.^1^, ^11^, ^13^, ^22^, ^23^, ^24^, ^25^ Finally, we constructed the network of DEPs and their involved functions by String web‐based platform (Figure 3E). Similar to the enrichment results of Metascape, complement system, lipid metabolism and the interaction between IGF and IGFBPs play essential roles in the process of infection, deserving further explorations.

### 3.4 The plasma protein prediction combination of the mortality of patients with COVID‐19

**FIGURE 4:**
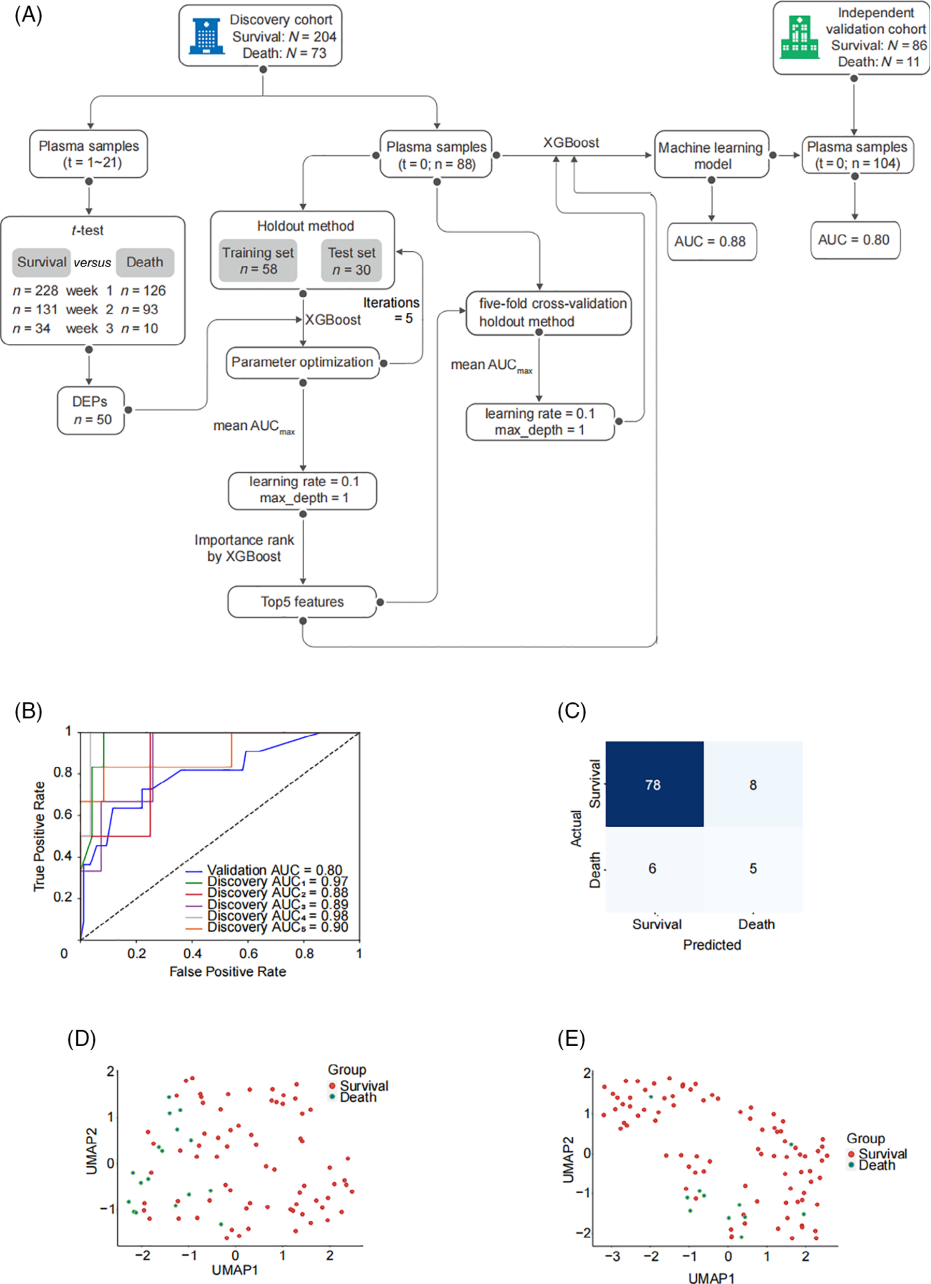
Identification of protein classifier to distinguish 28‐day survival and death groups using a XGBoost strategy. (A) The workflow to build a protein classifier for classification of 28‐day survival and death groups of COVID‐19 patients, including feature selection, parameter optimization and predictive model validation. (B) The receiver operating characteristic curves of the internal validation dataset across five‐fold cross‐validation and independent validation cohort using the five‐protein classifier. (C) The confusion matrix of the five‐protein classifier in the independent validation cohort. (D) The Uniform Manifold Approximation and Projection (UMAP) analysis of the five proteins among different plasma samples of the discovery cohort. (E) UMAP analysis of the five proteins among different plasma samples of the independent validation cohort.

The machine learning model was generated by using the plasma samples collected within 24 h of patients' admission. And the whole workflow to develop a protein classifier was shown in Figure 4A. For the 56 DEPs, after excluding 6 immunoglobins for feature selection, the left 50 DEPs were used to optimize learning rate and max_depth. The DEPs with the maximum value of the mean AUCs in the internal test dataset among five‐fold cross‐validation were selected to rank by relative importance. The top 5 protein features by importance were chosen to build the final classifier, namely CRP, extracellular matrix protein 1 (ECM1), IGF‐binding protein complex acid labile subunit (ALS), E3 ubiquitin‐protein ligase HECW1 and phosphatidylcholine‐sterol acyltransferase (LCAT). The relative importance of each factor was 0.155, 0.145, 0.113, 0.077 and 0.068 in order (Figure S2). According to the DEPs analysis, the five proteins showed different upward and downward trends in the whole course of disease. The expression of CRP was obviously up‐regulated in the death group during the first and second weeks after the patients' admission (Figures S3 and S4). For HECW1 and LCAT, they were both down‐regulated during the first week (Figure S3). ALS and ECM1 were also down‐regulated in the death group, but the changes happened during the second week (Figure S4).

Then, five‐fold cross validation was carried out using the whole discovery dataset with the five features to build the best model. The ROC curves of the internal validation dataset across five‐fold cross‐validation using the five‐protein model were shown in Figure 4B. Afterwards, we further optimized the parameters to make the model better. Ultimately, the best prediction model was constructed, with the mean AUC of 0.88.

To verify the prediction efficacy and universality of the five‐protein classifier, we collected plasma samples from Zhongshan Hospital to be the independent validation cohort. In this cohort, the AUC of the classifier was 0.80 (Figure 4B), certifying its excellent clinical potential and repeatability. The precision of the classifier in the independent validation cohort was evaluated by the confusion matrix (Figure 4C), showing its sensitivity and specificity are 92.9% and 38.5%, respectively.

Furthermore, uniform manifold approximation and projection (UMAP) analyses of the five DEPs were conducted in the two groups among all the patient plasma samples to visualize the prediction value of the five‐protein classifier. The results showed it can cluster the survival and dead patients in both discovery and validation groups (Figure 4D,E).

### 3.5 Predictive values of the existing factors and scores to predict 28‐day mortality

To compare the prediction efficiency of the five‐protein classifier with other biomarkers in predicting mortality at the early stage of the COVID‐19, we collected the data of CRP, ages, the absolute value of lymphocyte and neutrophil, NLR, the 4C mortality and E‐CURB65 scores on the first admission day of the patients in discovery and validation groups and respectively calculated the AUCs of these factors in predicting mortality. In discovery group, the AUCs of the indicators to predict mortality were as follows: CRP 0.67 [(95% CI: 0.60–0.75), *p* < 0.001], neutrophil count 0.69 [(95% CI: 0.62–0.77), *p* < 0.001], lymphocyte count 0.66 [(95% CI: 0.58–0.73), *p* < 0.001], NLR 0.74 [(95% CI: 0.67–0.80), *p* < 0.001], age 0.62 [(95% CI: 0.55–0.69), *p* < 0.01], the 4C mortality score 0.74 [(95% CI: 0.68–0. 80), *p* < 0.001] and E‐CURB65 score 0.78 [(95% CI: 0.72–0.85), *p* < 0.001] (Table 2). In the independent validation group, age, CRP and lymphocyte count showed no significant differences between dead patients and survivors. The AUCs of other indicators were as follows: neutrophil count 0.71 [(95% CI: 0.54–0.88), *p* < 0.05], NLR 0.72 [(95% CI: 0.58–0.85), *p* < 0.05], the 4C mortality score 0.77 [(95% CI: 0.60–0.94), *p* < 0.01] and E‐CURB65 score 0.80 [(95% CI: 0.69–0.92), *p* < 0.001] (Table 2).

**TABLE 2:** Diagnostic performance analysis of indicators and severity score for COVID‐19 patients. The diagnostic efficiencies of the indicators with no significant difference in predicting the outcome are not further explored.

	Discovery group				Validation group					
Prediction of mortality	Cut‐off value	AUC (95%CI)	Sensitivity (%)	Specificity (%)	Youden index	Cut‐off value	AUC (95%CI)	Sensitivity (%)	Specificity (%)	Youden index
Age (years)	69	0.62** (0.55–0.69)	44.61	78.08	0.23	‐	0.60 (0.43–0.78)	‐	‐	‐
CRP (mg/L)	65	0.67*** (0.60–0.75)	60.11	68.57	0.29	‐	0.65 (0.50–0.80)	‐	‐	‐
Neutrophil (10^9^/L)	7	0.69*** (0.62–0.77)	70	65.28	0.35	6.35	0.71* (0.54–0.88)	66.28	72.73	0.39
Lymphocyte (10^9^/L)	0.73	0.66*** (0.58–0.73)	50.25	73.61	0.24	‐	0.64 (0.47–0.80)	‐	‐	‐
NLR	14.22	0.74*** (0.67–0.80)	78.61	61.11	0.40	7.3	0.72* (0.58–0.85)	63.95	81.82	0.46
4C score	3	0.74*** (0.68–0. 80)	97.26	40.20	0.37	4	0.77** (0.60–0.94)	63.64	89.53	0.53
E‐CURB65 score	3	0.78*** (0.72–0.85)	56.16	89.22	0.45	2	0.80*** (0.69–0.92)	100	44.19	0.44
The classifier	‐	0.88	‐	‐	‐	‐	0.80	92.9	38.5	0.31

* *p* < 0.05;

** *p* < 0.01;

*** *p* < 0.001.

## 4 DISCUSSION

Since 2022, Omicron variants have become the main strain around the world, which have been proved to be more infectious and less toxic, always leading to mild symptoms.^26^, ^27^ However, epidemiological studies warned that elderly patients who are with other comorbidities and older than 65 are at high risk of developing into severe/critical after being infected with SARS‐CoV2 which means their risks of death are significantly increased while the quality of life is worsened.^28^ Therefore, in the wave of COVID‐19 from December, 2022 to January, 2023 in China, setting the mortality within 28 days of admission of the patients with different severities (mild, moderate, severe and critical) as clinical outcome, we collected plasma samples at different time points from the first day to the 21st day after the patients' admission and conducted proteomics analysis. Then, we collected plasma from the other medical centre for verifying the performance of the results. Finally, we determined a classifier consisting of five proteins which can effectively predict the clinical outcome of the patients diagnosed with COVID‐19 using trace plasma samples collected within 24 h of patients' admission.

In our study, we found the plasma proteins of survival and death groups presented consecutive changes at different time points after the admission. In the first week, the most proteins were expressed differently between the two groups, meaning the early changes of the plasma proteins can effectively predict the clinical outcome. As a result, our study further indicates the early plasma proteins changes should be concerned in clinical practice to guide the corresponding treatments. In addition, there were also persistent DEPs. For example, there were eight identical DEPs between the first and second weeks and one identical DEP between the first and third weeks. This phenomenon needs further exploration, may contributing to realize precise identification of the high‐risk patients.

We determined the pathophysiological reactions which play important roles in SARS‐CoV2 infection by pathway enrichment analysis and network of protein functions. There are complement system activation, regulation of IGF transport and uptake by IGFBPs, assembly, remodelling and clearance of plasma lipoprotein and binding and uptake of ligands by scavenger receptors, which are consistent with previous findings. A number of studies emphasized the activated acute phase proteins and complement system are closely associated with the severity of COVID‐19, for example, previous researches showed that hyperactivation of complement and coagulation systems can exacerbate the clinical symptoms of COVID‐19 patients.^29^, ^30^ Besides, scavenger receptor is a kind of pattern recognition receptor on the membrane of macrophages and influence lipid metabolism. Generally speaking, macrophages take part in the plasma lipids clearance while steroid hormone also regulates the functions of macrophages.^31^, ^32^ These findings demonstrated the relationship between severe/critical COVID‐19 and abnormal lipid metabolism.^33^ We also observed the interaction between IGF and IGFBP promoted the development of COVID‐19. IGF is indispensable in regulating the functions of growth factor, stimulating mitosis, inducing differentiation and promoting the expressions of different cell functions. IGFBP can bind to IGF, forming an inactive complex in the blood circulation. A Greek study also implied the connection between low IGF1 and poor outcomes of COVID‐19 patients.^34^

The prediction classifier of our research consists of five proteins. The early warning role of the up‐regulation of CRP in assessing the severity and predicting the prognosis of COVID‐19 has been repeatedly demonstrated,^35^, ^36^, ^37^, ^38^, ^39^ confirming its importance in clinical application. E3 ubiquitin‐protein ligase is a critical enzyme in ubiquitination^40^ associated with the progression of various inflammations^41^ and cancers.^42^ Ubiquitination and deubiquitination play important roles in SARS‐CoV‐2 infection.^43^ Besides, the low expression of HECW1, a kind of E3 ubiquitin‐protein ligase, has been proved to indicate the poor prognosis of clear cell renal cell carcinoma,^44^ suggesting its potential role in future study. LCAT is the central enzyme in the extracellular metabolism of plasma lipoproteins,^45^ having an important status in lipid metabolism. Previous studies also discovered that LCAT was down‐regulated in COVID‐19 patients and may be predictive of non‐survival in the patients,^46^, ^47^ due to the impaired liver function related with infection. ALS and ECM1 were down‐regulated in the second week, which is worth further exploration. In conclusion, the plasma protein biomarkers found in our study implies the close relationship between severe/critical SARS‐CoV2 infections and metabolic disorders, providing new directions for our future work.

In addition, we also estimated the prediction values of other biomarkers and scores used in clinical practice to further prove the superiority of the novel protein classifier. In results, 4C mortality and E‐CURB65 scores showed relatively good efficacy in predicting mortality in both discovery and validation groups. However, due to the limitations of traditional statistical analysis, it is difficult to further promote the prediction values of the score systems, which indicates novel technologies are required to construct prediction models with better effects. In recent advancements in medical research, machine learning/deep learning has proved to be a more powerful tool for prediction tasks. Obviously, the prediction efficiency of the five‐protein classifier developed by proteome profiling and machine learning is superior to the two comprehensive scores because of its higher AUCs in predicting the clinical outcome of the patients diagnosed with COVID‐19 in both discovery and independent validation cohorts. Therefore, the five‐protein classifier is believed to be a potential tool in future clinical use to realize the personalized treatments of patients in different conditions.

There are also flaws in this study. First, because of the management regulations related to COVID‐19 and the impact of hospital inpatient load, most of the enrolled patients had received pre‐hospital treatments (including antibiotic and antiviral treatments), which may influence the expressions of the plasma proteins and cause biases. Second, even though we tried our best to match several aspects of the two groups of patients, including the age, gender, comorbidities and severity of the disease, the sex, age ratios and the mortality rate in the validation group were not strictly controlled to be consistent with those in discovery group. This mismatch can lead to confounding bias and affect the validation results. To further optimize this research, in the future, we hope to conduct in‐depth study in conjunction with other medical centres to expand the research scale and control the baseline features of discovery and validation groups to explore deep molecular mechanisms.

## 5 CONCLUSIONS

In a word, we demonstrated the important role of acute inflammation in severe/critical COVID‐19 and developed a classifier containing CRP, ECM1, HECW1, ALS and LCAT by using proteomics and machine learning technologies. This five‐protein classifier shows greater advantages in early prediction of the mortality within 28 days of COVID‐19 patients' admissions than other existing biomarkers and score systems. Besides, we also collected an independent patient cohort to verify the prediction efficacy of this model and found the results showed good reproducibility. More importantly, the whole test needs only 1 μL early plasma from the patients with COVID‐19, with less trauma. Therefore, we believe the classifier developed in our study has enormous potential in predicting the severity and prognosis of the diagnosed patients with COVID‐19, which contributes to guide the clinical treatments and reduce the incidence of adverse outcomes in high‐risk patients. Also, this work provides abundance proteomics resource that may inspire the whole research community and deserves further exploration in the future.

## AUTHOR CONTRIBUTIONS

Yifei Zeng and Feng Xu were involved in the design of the study. Yifei Zeng collected all the plasma samples and patients' data in discovery group and carried out statistical analysis and wrote the first draft of the manuscript. Yufan Li collected the plasma samples in validation group and provided the patient cohort. Huidan Lu, Siyi Lin, Wenting Zhang, Wanying Zhang, Lexin Xia and Huiqun Hu contributed to collect the samples and medical data and offered some statistical advice. Yuanlin Song and Feng Xu further revised the results and manuscript. All authors have approved the final manuscript. The corresponding authors confirm that all listed authors meet authorship criteria.

## FUNDING INFORMATION

This research was funded by the National Natural Science Foundation of China (Grant No. 81970004 and 82241046), Binjiang Institute of Zhejiang University (ZY202205SMKY006) and Qianjiang Distinguished Scholar Program from Hangzhou City.

## CONFLICT OF INTEREST STATEMENT

The authors declare no conflicts of interest.

## Supporting information


**Data S1:** Supporting Information.

## Data Availability

The data that support the findings of this study are available from the corresponding author upon reasonable request.
