# Supplementary material for: Proteome analysis develops novel plasma proteins classifier in predicting the mortality of COVID‐19
Source: Cell Prolif. 2024 Feb 25;57(7):e13617. doi: 10.1111/cpr.13617 (PMC11216943; doi:10.1111/cpr.13617)
Supplement: Supplementary file 1 — Data S1: Supporting Information. [file CPR-57-e13617-s001.docx]

**
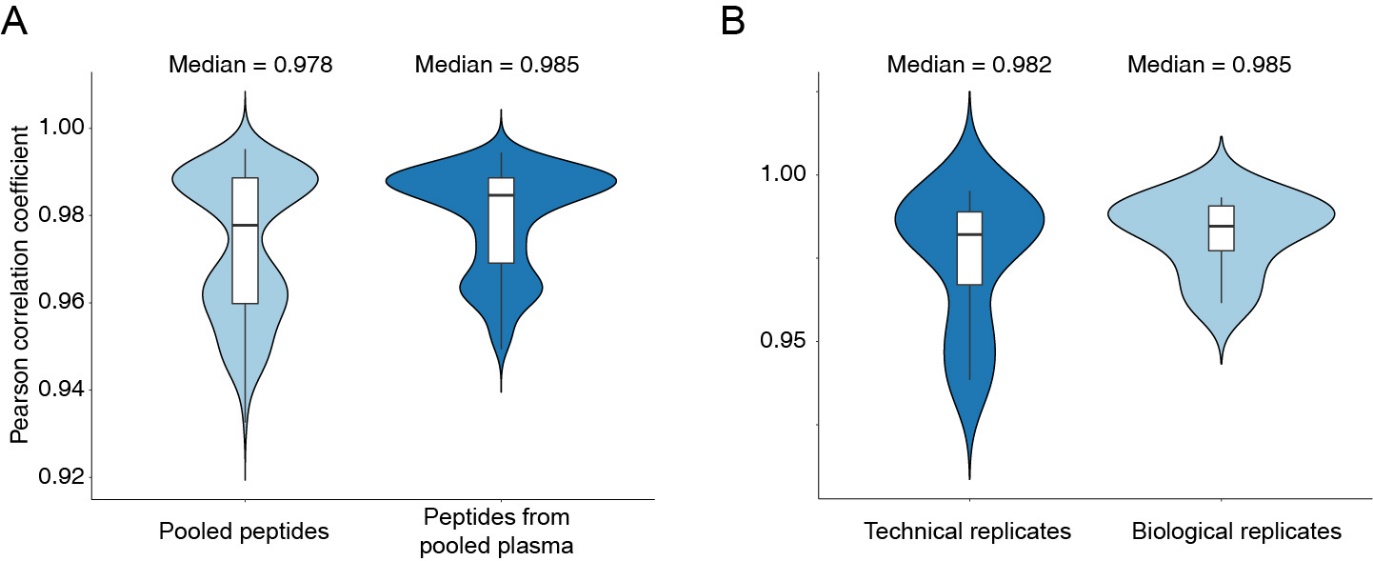
**

**Additional Figure 1 Quality control for proteomic data of validation cohort.**

1. Pearson correlation coefficients of protein expression among pooled peptides and peptides from pooled plasma, respectively, from both discovery and validation cohort. (B) Pearson correlation coefficients of protein expression among biological and technical replicates, respectively, from the validation cohort.


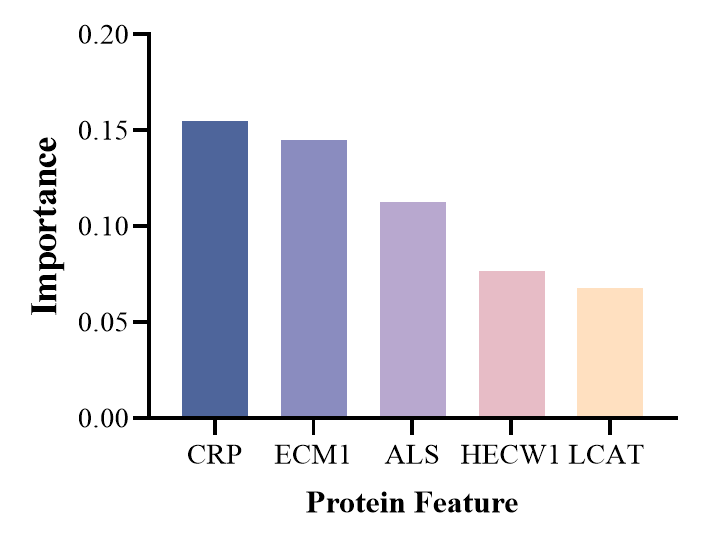


**Additional Figure 2 The relative importance of the five protein features.**

The five features, namely CRP, ECM1, ALS, HECW1 and LCAT, were ranked by the relative importance. CRP: 0.155; ECM1: 0.145; ALS: 0.113; HECW1: 0.077 and LCAT:0.068.


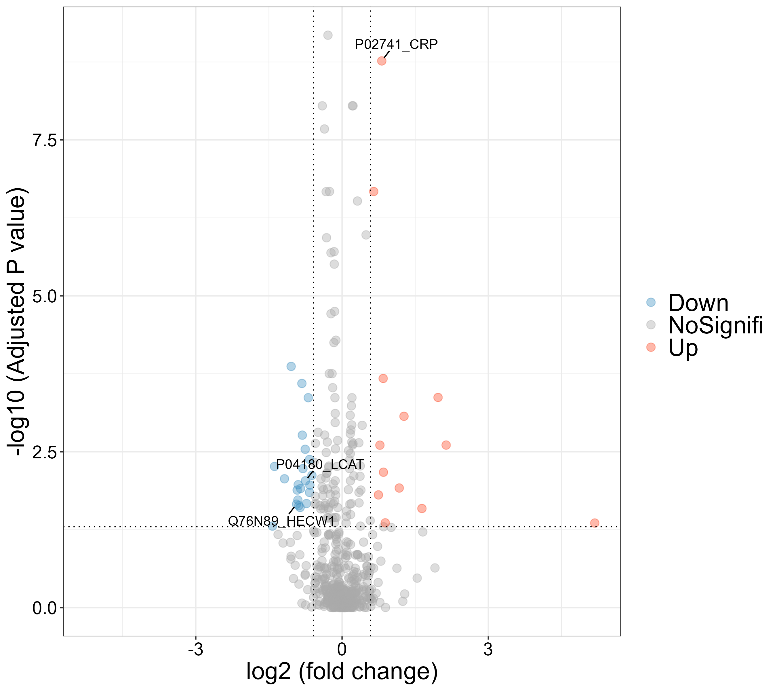


**Additional Figure 3 The up and down regulations of CRP, LCAT and HECW1 in the first week.**

The figure showed CRP was up-regulated while LCAT and HECW1 were down-regulated in the first week.


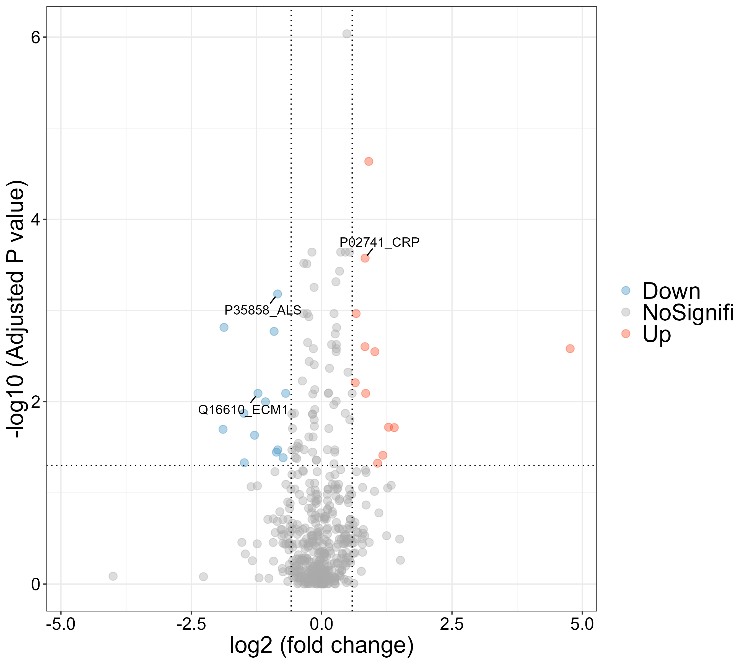


**Additional Figure 4 The up and down regulations of CRP, ALS and ECM1 in the second week.**

The figure showed CRP was up-regulated while ALS and ECM1 were down-regulated in the second week.
